# Financial Decision-Making in Neurological Patients

**DOI:** 10.3390/brainsci12050529

**Published:** 2022-04-21

**Authors:** Laura Danesin, Andreina Giustiniani, Giorgio Arcara, Francesca Burgio

**Affiliations:** IRCCS San Camillo Hospital, 30126 Venezia, Italy; andreina.giustiniani@hsancamillo.it (A.G.); giorgio.arcara@hsancamillo.it (G.A.); francesca.burgio@hsancamillo.it (F.B.)

**Keywords:** financial abilities, mild cognitive impairment, Parkinson’s disease, stroke, decision-making

## Abstract

Financial abilities (FA) are a multi-dimensional domain comprising a wide range of conceptual, pragmatical, and judgmental skills ranging from basic abilities, such as bill payment, to high level abilities, such as financial decision-making (FDM). Preserved FDM abilities include the capacity to recognize fraud attempts, and they are fundamental for a person’s independence. Previous studies have reported decreased FDM in older adults and in patients with mild cognitive impairment (MCI), who consequently become more susceptible to fraud attempts. However, FDM has scarcely been investigated in other neurological populations, and it is unclear whether FDM may be predicted by more basic FA. The aim of the present study was to investigate FDM across patients with MCI, Parkinson’s disease (PD), or stroke, as well as healthy controls (HC), and to explore to what extent FDM could be inferred by other FA. We collected FDM and FA performances using the NADL-F short battery. Performances in the NADL-F short subtests were compared among groups. Additionally, the relationship between the scores at the FDM subtest and the performance obtained in other financial subtests of the NADL-F short were investigated for each group of participants. MCI patients performed worse than HC in FDM and in several FA domains. Conversely, FDM was relatively preserved in our sample of PD and stroke patients. In HC, FDM was associated with numeracy and financial knowledge applied to everyday situations, whereas this was true with some basic FA in both MCI and PD patients. No significant association was observed in stroke patients. Our results suggest that FDM is a complex ability, only partially inferable from other FA.

## 1. Introduction

Financial abilities (FA) are defined as the capacity to manage money and financial assets in ways that meet a person’s needs, and that are consistent with their values and self-interest [1]. FA are a multi-dimensional domain comprising a wide range of conceptual, pragmatical, and judgmental skills [2], relevant for daily activities and related to managing finances, to different levels. Among these, we can distinguish some more basic abilities strictly related to numerical skills (e.g., handling money, making small purchases, paying bills, counting currencies, and reading numbers) and higher-level abilities (e.g., knowledge about financial concepts and financial decision-making) requiring a more complex interplay between basic financial skills and numerical abilities, and other higher-order cognitive functions (e.g., planning). A high-level FA that has recently gained the attention of the scientific community is the one regarding financial decision-making (FDM), which concerns making financial choices under uncertainty, when the potential gains or losses of the choice and their probabilities are not clear, and when the scenario may even hide a fraud. In the last decade, several studies have examined decision-making abilities across the human lifespan, reporting that older adults might encounter some difficulties when having to deal with decisions in everyday contexts, especially financial ones [3,4,5,6]. Difficulties in FDM may have detrimental consequences on a person’s autonomy and wellbeing, leading to poverty or financial exploitation [7,8]. Impaired FDM have been found in both healthy older adults and neurological patients [5,9]. Indeed, almost 5% of older adults become victims of financial fraud, with an annual incidence of 2.7% [10,11], leading to losses of approximately $2.9 billion annually [12]. Patients with neurological diseases are even more susceptible to fraud attempts [9]. In particular, at some level, impairments in FDM and in FA are related to the degree of cognitive decline [13]. Indeed, some previous studies have reported that difficulties in FDM are associated with deficits in memory, visuospatial abilities, and executive functions in older adults [5]. General cognition, numeracy, attention, prospective memory, and executive functions are related to FDM in patients with mild cognitive impairment (MCI), Alzheimer’s disease’s (AD) [9,14,15,16], and Parkinson’s disease (PD) [17]. Similarly, an association between cognitive deficits and FA has been found in many pathological populations [15,18,19,20]. These previous studies clarified the association between cognitive deficits and FDM, as well as between cognitive deficits and FA, but questions remain whether, regardless of cognitive impairment, FA performances might predict FDM in both healthy subjects and patients with neurological diseases. In other words, it is not clear whether FDM relies on basic FA and, if so, which FA are mostly associated to FDM. Additionally, to the best of our knowledge, no study has investigated FDM in cerebrovascular diseases such as stroke. Stroke is the third most common cause of adult disability [21] and it is often associated with long-term impairments in cognitive functioning [22] and in everyday activities [23].

Exploring the possible association between FDM and FA might have useful impacts in patients’ and caregivers’ daily life. Indeed, knowing the exact characteristics of the deficits affecting patients can support caregivers’ choices with respect to patients’ financial management. This is even more crucial in the legal context, where FDM is often inferred from general surveys, heavily relying on the person’s self-awareness of their own capacities [13]. Additionally, no studies have compared deficits in FA and FDM across different pathologies and healthy subjects using the same instrument. This would minimize differences due to the tool used for the assessment, rather than due to the intrinsic characteristics of the studied population. Investigating FDM in neurological patients by using the same instrument might shed further light on their cognitive conditions and on how the multidimensional construct of FA declines.

Therefore, the aim of the present study was to investigate FDM across three neurological populations (i.e., MCI, PD, and stroke) and healthy elderly individuals, by using a specific subtest of the NADL-F short battery [24], and to explore to what extent FDM could be related to other FA. To this aim, performances at the NADL-F short subtests were compared among groups. Additionally, the relationship between the scores at the FDM subtest (i.e., financial judgments) and the performance obtained in other financial subtests of the NADL-F short were investigated for each group of participants.

## 2. Materials and Methods

### 2.1. Participants

Participants’ demographics are reported in Table 1. We analyzed data from 303 participants divided into four groups: 104 MCI patients, 62 PD patients, 53 stroke patients (34 right and 19 left stroke), and 84 healthy controls (HC), recruited at IRCCS San Camillo Hospital (Venice, Italy). The dataset consisted of data collected from 2014 to 2021. The HC (55 females and 29 males) had a mean age of 70.45 years (SD = 8.01). MCI patients (46 females and 58 males) had a mean age of 75.05 years (SD = 6.69). PD patients (23 females and 39 males) had a mean age of 68.48 years (SD = 8.77). Right stroke patients (8 females and 26 males) had a mean age of 67.00 years (SD = 11.51). Lastly, patients with left stroke (10 females and 9 males) had a mean age of 64.58 years (SD = 11.69). Participants underwent a neuropsychological assessment at the time of the data collection and were diagnosed by experienced neurologists according to standard clinical criteria [25,26,27]. In particular, MCI patients had multi-domain cognitive impairments, i.e., with difficulties in memory and in at least another cognitive domain [25]. On the other hand, PD patients without clinically relevant cognitive impairments (i.e., MMSE score > 24 and the absence of subjective complaints of cognitive difficulties) were enrolled. Lastly, stroke patients had lesions affecting heterogeneous brain regions.

HC were recruited among patients’ family members or through adverts. All HC were autonomous and had no developmental learning disorders or relevant pathologies that could affect their cognitive performance at the time of the assessment.

Exclusion criteria for all participants were history of other neurological or psychiatric diseases, presence of dementia, severe verbal comprehension deficits, not corrected to normal visual deficit, and the inability to give informed consent.

All participants took part in the study on a voluntary basis and gave their informed consent according to the Helsinki Declaration. The study was approved by the Ethics Committee of Venice and IRCCS San Camillo Hospital (Venice, Italy), reference number 2016.07.

### 2.2. Materials

The whole sample performed the NADL-F short battery [24] and the Mini-Mental State Examination test [28].

The NADL-F short battery includes seven subscales investigating separate domains of FA. In particular, the following five subtests were used for the assessment of basic FA:

- Counting Currencies (maximum score = 3) evaluates whether the participant is familiar with the euro currency and is able to perform simple mental calculations, analogous to those involved in simple cash transactions but in a simplified setting.
- Reading Abilities (maximum score = 3) investigates whether the participant is able to deal with written information about money in everyday life situations.
- Item Purchase (maximum score = 4) evaluates the ability of the participant to deal with operations (e.g., calculations, keeping in mind the relevant information) that are necessary to make cash transactions during shopping in real life.
- Bill Payments (maximum score = 2) evaluates knowledge regarding managing bills.
- Percentages (maximum score = 3) investigates whether the participant is able to perform the mental calculations needed in percentages in real-life contexts.

Two subtests were used to assess high level FA, namely financial concepts and financial judgments. Financial Concepts (maximum score = 6) assesses the participant’s knowledge of financial concepts that are relevant in the Italian cultural context, whereas Financial Judgments (maximum score = 2) investigates whether the participant is able to make meaningful financial judgements and detect fraudulent behaviors. In particular, the Financial Judgments subtest was used to assess FDM.

### 2.3. Statistical Analysis

The Kolmogorov–Smirnov test was used to test normality. As demographic data (age, education, and sex) were not normally distributed, Kruskal–Wallis or Chi-square tests were used to investigate the differences between HC and patients.

To investigate the differences in FDM and FA between groups and to explore the possible association between FA domains and FDM, we firstly performed separate one-way ANOVA tests for each of the seven domains of NADL-F short, with the score as the dependent variable and group (five levels: MCI, Left Stroke, Right Stroke, PD, HC) as the factor. Post hoc analyses were conducted by applying the Tukey’s HDS test, *p*-values were adjusted with Bonferroni correction to take into account the possibility of inflated type I errors, and a *p* < 0.01 was considered significant. Afterwards, to investigate whether performances in FA could predict the performance in FDM, we performed a forward stepwise multiple regression analysis adjusted for age, sex, and education to examine the association between FDM and the other domains of FA for each group separately. Lastly, performances above/below the cut-off values were calculated for each NADL-F short subtest based on normative data [24], and the co-occurrence of FDM and FA deficits in the different groups was explored by means of contingency tables and the Chi-square test. To take into account the possibility of inflated type-I errors, Bonferroni correction for multiple comparisons was applied to significant *p*-values. In particular, Pearson’s Chi-square was used as a test of independence; the model in which the occurrence of deficits in FDM was independent from the occurrence of deficits in other FA domains was considered as null. Albeit partially overlapping with the analysis on the raw score, this analysis using cut-off values based on normative data provides important additional information for clinicians, as the cut-offs are those threshold values used in the clinical setting to infer an impairment to the function measured by the task. In contrast, analyses on raw scores can also capture significant but small effects, which are negligible in clinical practice.

## 3. Results

Table 1 shows participants’ demographic characteristics. Comparison analyses revealed significant differences between groups for age (χ^2^ = 42.45; *p* < 0.001), educational level (χ^2^ = 14.54; *p* = 0.006), and sex (χ^2^ = 22.04; *p* < 0.001). Patients with MCI were generally older, whereas stroke patients had a lower education level compared with the other groups. Moreover, a significant difference between groups was observed for MMSE performances (χ^2^ = 35.51; *p* < 0.001), with MCI, right stroke, and left stroke patients showing lower MMSE scores compared with HC.

**Figure 1:**
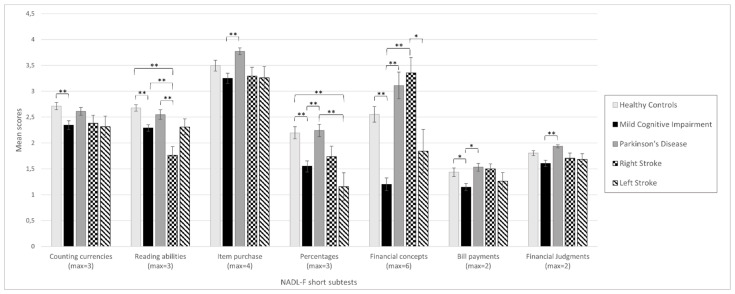
Mean scores at each NADL-F short subtest across all of the groups. The maximum score that can be obtained for each subtest is reported in parentheses. Error bars show standard errors of the means. Asterisks represent significant differences (* *p* < 0.01; ** *p* < 0.001).

The NADL-F short performances for each group are presented in Figure 1 (see also Table S1 in the Supplementary Materials). ANOVA revealed that there were statistically significant differences between groups in all of the seven NADL-F short subtests (Counting Currencies: F(4, 298) = 5.81, *p* < 0.001; Reading Abilities: F(4, 298) = 14.36, *p* < 0.001; Item Purchase: F(4, 298) = 5.02, *p* = 0.001; Percentages: F(4, 298) = 9.61, *p* < 0.001; Financial Concepts: F(4, 298) = 22.12, *p* < 0.001; Bill Payments: F(4, 298) = 4.53, *p* = 0.001; and Financial Judgments: F(4, 298) = 4.72, *p* = 0.001). Table S2 shows the results of Tukey’s HDS post hoc correction for multiple comparisons. MCI performed worse than HC in all of the NADL-F short subtests. However, after having corrected for Bonferroni multiple comparisons, the differences in Item Purchase and Financial Judgments were no longer significant. Right stroke patients showed lower performances in Counting Currencies and Reading Abilities compared to HC, with only the latter surviving after Bonferroni correction. Left Stroke patients performed worse than HC only in Reading Abilities and Percentages, with only the first one surviving Bonferroni correction. Conversely, no differences were observed between HC and PD across the seven subtests of NADL-F short.

Table 2 presents the results of the stepwise multiple regression for FDM in each group, adjusted for age, education, and sex. The domain of Financial Judgments was positively associated with Bill Payments (*p* < 0.001) and Item Purchase (*p* = 0.018) in HC and MCI patients, respectively. On the other hand, the domain of Counting Currencies was positively associated with FDM in PD patients, whereas Item Purchase was negatively associated with FDM (Counting Currencies: *p* = 0.015; Item Purchase: *p* = 0.028). No significant association between NADL-F short subtests and FDM was observed for right nor left stroke patients.

Chi-square tests were used to compare the occurrence of deficits in the FDM subtest and the other domains of NADL-F short in each group (Table 3 and Table 4). In HC, deficits in Percentages and in Bill Payment domains were significantly associated with deficits in FDM; however, only the presence of deficits in Bill Payments remained significantly associated with the occurrence of deficits in FDM after Bonferroni correction. On the other hand, impairments in Financial Concepts were associated with FDM deficits in MCI patients, but this association did not survive Bonferroni correction for multiple comparisons. No significant association was observed in the other groups.

## 4. Discussion

The aim of the present study was to investigate FDM in different neurological populations, so as to explore which abilities among FA are mostly associated with FDM and whether a deficit in FDM can be predicted from a deficit in basic FA. We found a trend towards decreased FDM in our sample of MCI patients, whereas this ability was relatively preserved in PD and stroke patients. Previous studies reported impairment in FA in patients with MCI [15,20,29]. Here, we expand this previous finding reporting that the performance in FA may predict FDM performance in healthy subjects, in MCI patients, and in PD patients, and that, similarly, impairment in FA is associated with a deficit in FDM in healthy subjects and MCI. However, it should be noted that only specific FA are associated with FDM. Namely, among preserved financial abilities, only performances in Bill Payment, Item Purchase, and Counting Currencies exhibited an association with FDM in healthy subjects, MCI patients, and PD patients, respectively. On the other hand, when a deficit emerged in FDM, this was associated with Percentages and Bill Payment in healthy subjects, and with Financial Concepts in MCI patients.

Despite, at first glance, the presence of an association between impaired FDM and FA in healthy subjects appearing to be counterintuitive, it is in line with previous studies reporting fragile FA in cognitively preserved older adults [5]. The finding of an association between percentages and fraud recognition is consistent with a recent study reporting a relationship between numeracy and FDM in older adults [30]. On the other hand, the association between bill payment and FDM in healthy subjects needs further explanation. Indeed, the ability to pay bills requires people to have a certain level of financial literacy and to be able to apply it in daily living. Financial literacy has been recognized as a protective factor against financial exploitation from a younger age [31], and has been recently related to a higher capability to detect fraud attempts [32]. Therefore, we can argue that, assuming no other cognitive deficits, the presence of a certain financial literacy required for Bill Payment is also associated with FDM. However, previous studies have suggested that cognitive changes related to normal aging, such as those in working memory, could also affect decision-making abilities [3]. Despite not exhibiting clinically relevant cognitive impairments, our sample showed some cognitive difficulties (measured with the MMSE) due to normal aging that might have affected the performance in FDM.

We found that MCI patients exhibited lower performances in several FA domains compared with the healthy controls, as well as decreased FDM. These results are in line with previous works reporting financial difficulties in these patients [29]. Such difficulties may then reflect also on FDM. In particular, the ability to make everyday purchases was positively associated with fraud recognition, whereas difficulties in financial concepts were associated with deficits in FDM in these patients. A previous study reported that intact basic FA, such as the ability to make everyday purchases, only have minimal effects on the ability to detect fraud [32]. Nowadays, fraud complexity has increased alarmingly; therefore, even if efficient basic FA can reduce an individual’s exposure to be a target for fraud, alone, they are not enough to spot fraudulent behavior [32]. Moreover, a person’s financial knowledge seems to not completely account for the ability to recognize fraud attempts. Indeed, even though difficulties in financial concepts were initially associated with deficits in FDM, this did not survive multiple comparison correction, thus indicating that other abilities might be involved in FDM. For instance, previous studies have suggested that a person’s self-awareness of their own abilities may be another relevant factor for the efficient detection of fraud [30]. Furthermore, particularly in MCI patients, a person’s specific spectrum of cognitive deficits may play a role in FA and FDM. For example, Benavides-Varela and colleagues [29] suggested that, when executive functions breakdown, the emotional processes normally related with decision-making [33] prevail over the cognitive ones, thus negatively influencing the individual’s ability to correctly assess the consequences of decisions [29]. In line with this, we might speculate that, as cognitive functions are also involved, basic FA and higher-level FA alone are probably not enough to predict FDM, but when these abilities are impaired, the probability of the occurrence of FDM deficits is higher.

PD patients showed overall good performances in FA and FDM, similar to those of the healthy controls. Indeed, our PD patients did not show cognitive impairments. A previous study reported impaired FA in patients with PD when they exhibited MCI or dementia [18]. However, these impairments may have been related to concurrent cognitive difficulties rather than to PD pathology per se. Therefore, we suggest that, in the absence of cognitive deficits, PD patients retain FDM. In particular, in this group, FDM was associated with basic FA, such as Counting Currencies and Item Purchase. Of note, these two tasks involve simple operations using money. Therefore, as seen in healthy older adults [30], it could be that numeracy is associated with FDM in PD patients with normal cognition.

Right stroke patients exhibited difficulties in Counting Currencies and Reading Abilities, whereas left stroke patients exhibited difficulties in Reading Abilities and Percentages. However, in both left and right stroke patients, none of the basic FA was associated with FDM. Difficulties in calculation in right and left stroke patients have been widely described in the literature, where they have been ascribed mostly to numerical deficits in left-hemisphere stroke patients and to spatial deficits in right-hemisphere stroke patients [34]. However, despite some deficits in FA, our patients did not exhibit deficits in FDM. Several reasons might account for the absence of difficulties in such abilities. First, these patients were impaired in basic FA such as Reading Abilities and Counting Currencies, but preserved relatively higher level FA such as Financial Concepts. As stated above, for MCI patients, it is possible to argue that, with higher level FA being preserved, patients are still able to detect fraud attempts. Secondly, from a cognitive point of view, fraud recognition lays, to some extent, on abstract reasoning and executive functioning [35]; we might argue that our patients compensated for the deficit in FA through relatively preserved reasoning ability and executive functions. In line with this hypothesis, a previous study investigating general decision-making capacities in stroke patients reported that these capacities strongly rely on executive functions and language comprehension [36]. However, there are some limitations that we have to acknowledge with respect to our sample of stroke patients, which might partially account for our results. Namely, we did not divide patients according to the site of the lesion, thus resulting in a highly heterogeneous sample and limiting the possibility to speculate about the possible mechanisms involved in FA deficits, as well as the relatively preserved processes that might account for the lack of deficits in FDM. Further studies taking into account the brain regions affected by stroke are needed to clarify this point. A study limitation that we have to acknowledge is that we did not assess specific cognitive domains that could have been involved in FDM, such as abstract reasoning and executive functions, and further studies would be needed investigating the relationship between FA, FDM, and other cognitive functions. Moreover, when considering the contingencies between deficits in FDM and impaired FA, we have to acknowledge that in some cases, the cell sizes were small, thus limiting the power of our analyses. Future studies should investigate this relationship by enrolling larger sample sizes.

Taken together, our results suggest that, despite being part of the same general framework, FDM is a complex ability only partially relying on basic FA. In particular, on the one hand, when FDM and FA are preserved, there is a certain association between FDM and specific basic FA (e.g., counting currencies), on the other hand when a deficit is present, that is, when patients’ performance is below the cut-off, this association changes and other basic FA (e.g., knowledge of financial concepts) appear to predict FDM performance. Importantly, when considering performance above or below cut-offs (which are the thresholds used in clinical practice), an association between FDM and FA emerged in a few cases; but, in most cases, having a deficit in FDM was not associated with a deficit in other FA. This may be due to the investigation of the co-occurrence of deficits (i.e., performance below cut-off) distinguished between overt impairments and acceptable performance, rather than the subtle effects that may be captured when using raw data and regressions. Moreover, this lack of association might be due to other processes intervening to compensate for the difficulties in FDM, such as self-awareness and emotional status [37,38]. This is particularly relevant in clinical and forensic practice, which often face the challenge of FDM evaluation and should not limit the assessment to numerical abilities and basic capacity to use money. In other words, neither the basic financial capacities nor the diagnosis per se, nor the neuropsychological assessment, are sufficient to infer FDM. Specific tests are needed to assess these abilities [20,37] and to avoid financial exploitation, supporting legal choices with respect to the patients’ financial management.

Additionally, regardless of the cognitive status and overall FA, FDM is often impaired in neurodegenerative diseases, whereas it might be relatively preserved in cerebrovascular patients. This knowledge could guide both clinicians’ and caregivers’ choices with respect to the financial management of patients, depending on the pathology.

Our results encourage further investigations to study FDM in other pathologies, as well as exploration through neuroimaging and behavioral studies of the brain regions and relative cognitive domains underlying this ability. Moreover, given their role in FDM, future studies should investigate how emotional and awareness factors are related to FDM and other FA.

## Figures and Tables

**Table 1:** Demographic characteristics, means, and standard deviations (SD) or number of participants.

	Healthy Controls	Mild Cognitive Impairment	Parkinson’s Disease	Right Stroke	Left Stroke	*p*-value
*N*	84	104	62	34	19	
Age, y (SD)	70.45 (8.01)	75.05 (6.69)	68.48 (8.77)	67.00 (11.51)	64.58 (11.69)	<0.001 ^a^
Education, y (SD)	12.81 (4.66)	10.87 (4.61)	11.68 (4.89)	9.85 (4.72)	9.47 (4.48)	0.006 ^a^
Male, *n* (%)	29 (34.52)	58 (55.77)	39 (62.90)	26 (76.47)	9 (47.37)	<0.001 ^b^
MMSE (SD)	27.83 (2.10)	25.15 (3.10)	27.03 (2.47)	25.57 (2.92)	26.10 (1.85)	<0.001 ^a^

^a^ Kruskal–Wallis test; ^b^ Pearson’s Chi-square test.

**Table 2:** Significant multiple regression models for FDM in each group. Bold indicates statistically significant values.

	B	SE B	β	*p*-Value	Partial Correlation
*Healthy controls*					
Constant	1.580	0.480		0.002	
Age	7.60 × 10^−5^	0.006	0.001	0.990	0.001
Education	−0.016	0.011	−0.169	0.148	−0.162
Sex	0.043	0.099	0.045	0.665	0.049
Bill payments	0.275	0.070	0.458	**<0.001**	0.405
*Mild Cognitive Impairment*					
Constant	0.883	0.775		0.258	
Age	−0.001	0.009	−0.009	0.928	−0.009
Education	0.020	0.013	0.152	0.127	0.153
Sex	0.254	0.120	0.206	**0.037**	0.208
Item purchase	0.139	0.058	0.232	**0.018**	0.236
*Parkinson’s disease*					
Constant	1.598	0.354		<0.001	
Age	0.007	0.004	0.251	0.057	0.251
Education	0.002	0.007	0.035	0.800	0.034
Sex	0.062	0.064	0.121	0.339	0.128
Counting currencies	0.163	0.065	0.403	**0.015**	0.319
Item purchase	−0.164	0.072	−0.348	**0.028**	−0.289

**Table 3:** Chi-square and *p* value relative to the association between participants’ deficits in FDM and in each other subtest of the NADL-F short. Significant *p*-values are reported in bold.

	N	Chi-Square	df	*p*	Contingency Coefficient
*Counting Currencies*					
Healthy controls	84	10.64	1	0.200	0.138
Mild cognitive impairment	104	00.66	1	0.415	0.080
Parkinson’s disease	62	00.29	1	0.587	0.069
Right stroke	34	20.24	1	0.134	0.249
Left stroke	19	00.01	1	0.943	0.016
Total	303	00.84	1	0.358	0.053
*Reading Abilities*					
Healthy controls	84	20.86	1	0.091	0.181
Mild cognitive impairment	104	10.58	1	0.209	0.122
Parkinson’s disease	62	00.50	1	0.481	0.089
Right stroke	34	10.05	1	0.306	0.173
Left stroke	19	00.90	1	0.342	0.213
Total	303	30.15	1	0.064	0.106
*Item purchase*					
Healthy controls	84	10.03	1	0.311	0.110
Mild cognitive impairment	104	00.38	1	0.538	0.060
Parkinson’s disease	62	00.22	1	0.641	0.059
Right stroke	34	10.39	1	0.238	0.198
Left stroke	19	00.35	1	0.554	0.135
Total	303	10.31	1	0.253	0.066
*Percentages*					
Healthy controls	84	40.45	1	**0.035**	0.224
Mild cognitive impairment	104	10.91	1	0.167	0.134
Parkinson’s disease	62	10.65	1	0.198	0.161
Right stroke	34	00.71	1	0.400	0.143
Left stroke	19	00.22	1	0.636	0.108
Total	303	70.83	1	0.005 *	0.159
*Financial concepts*					
Healthy controls	84	00.62	1	0.430	0.086
Mild cognitive impairment	104	30.95	1	**0.047**	0.191
Parkinson’s disease	62	00.46	1	0.499	0.086
Right stroke	34	00.65	1	0.419	0.137
Left stroke	19	00.65	1	0.419	0.182
Total	303	90.96	1	0.002 *	0.178
*Bill Payments*					
Healthy controls	84	150.31	1	<0.001 *	0.393
Mild cognitive impairment	104	00.60	1	0.438	0.076
Parkinson’s disease	62	00.21	1	0.641	0.059
Right stroke	34	00.32	1	0.573	0.096
Left stroke	19	00.35	1	0.554	0.135
Total	303	90.73	1	0.002 *	0.176

* *p*-values remained significant after Bonferroni correction.

**Table 4:** Number (%) of participants above/below the cut-off in the seven NADL-F short subtests for each group.

	Counting Currencies		Reading Abilities		Item Purchase		Percentages		Financial Concepts		Bill Payments		Financial Judgments	
	Above	Below	Above	Below	Above	Below	Above	Below	Above	Below	Above	Below	Above	Below
HC	82 (97.6)	2 (2.4)	63 (75.0)	21 (25.0)	73 (86.9)	11 (13.1)	74 (88.1)	10 (11.9)	81 (96.4)	3 (3.6)	71 (84.5)	13 (15.5)	70 (83.3)	14 (16.7)
MCI	87 (83.6)	17 (16.4)	40 (38.5)	64 (61.5)	86 (82.7)	18 (17.3)	79 (76.0)	25 (24.0)	66 (63.5)	38 (36.5)	84 (80.8)	20 (19.2)	70 (67.3)	34 (32.7)
PD	58 (93.5)	4 (6.5)	41 (66.1)	21 (33.9)	59 (95.2)	3 (4.8)	57 (91.9)	5 (8.1)	56 (90.3)	6 (9.7)	59 (95.2)	3 (4.8)	58 (93.5)	4 (6.5)
RS	28 (82.3)	6 (17.7)	9 (26.5)	25 (73.5)	30 (88.2)	4 (11.8)	26 (76.5)	8 (23.5)	32 (94.1)	2 (5.9)	33 (97.1)	1 (2.9)	26 (76.5)	8 (23.5)
LS	16 (84.2)	3 (15.8)	6 (31.6)	13 (68.4)	17 (89.5)	2 (10.5)	11 (57.9)	8 (42.1)	12 (63.2)	7 (36.8)	17 (89.5)	2 (10.5)	13 (68.4)	6 (31.6)
Total	271 (89.4)	32 (10.6)	159 (52.5)	144 (47.5)	265 (87.5)	38 (12.5)	247 (81.5)	56 (18.5)	247 (81.5)	56 (18.5)	264 (87.1)	39 (12.9)	237 (78.2)	66 (21.8)

## Data Availability

The data presented in this study are available upon request from the corresponding author. The data are not publicly available due to ethical reasons.

## Supplementary Materials

The following supporting information can be downloaded at: https://www.mdpi.com/article/10.3390/brainsci12050529/s1. Table S1: NADL-F performances across the sample groups, means, and standard deviations. Table S2: Tukey’s HDS post hoc correction for multiple comparisons.
