# Supplementary material for: Financial Decision-Making in Neurological Patients
Source: Brain Sci. 2022 Apr 21;12(5):529. doi: 10.3390/brainsci12050529 (PMC9139159; doi:10.3390/brainsci12050529)
Supplement: Supplementary file 1 [file brainsci-12-00529-s001.zip › brainsci-1629415-supplementary.pdf]

Financial decision-making in neurological patients

Danesin L., Giustiniani G., Arcara G., Burgio F.

Supplementary materials

Table S1. NADL-F performances across the sample groups, means and standard deviations

	Healthy controls	Mild Cognitive Impairment	Parkinson's Disease	Right stroke	Left stroke	*p-value*
Counting currencies	2.81 (0.45)	2.35 (0.84)	2.61 (0.61)	2.38 (0.92)	2.32 (0.88)	<.001
Reading abilities	2.69 (0.58)	2.28 (0.64)	2.61 (0.61)	1.76 (1.02)	2.16 (0.69)	<.001
Item purchase	3.62 (0.71)	3.25 (1.02)	3.77 (0.52)	3.29 (1.00)	3.26 (0.93)	.001
Percentages	2.27 (1.03)	1.55 (1.08)	2.24 (0.95)	1.74 (1.19)	1.16 (1.17)	<.001
Financial concepts	2.63 (1.36)	1.20 (1.26)	3.11 (2.03)	3.35 (1.74)	1.84 (1.83)	<.001
Bill payments	1.48 (0.75)	1.14 (0.72)	1.53 (0.59)	1.50 (0.56)	1.32 (0.67)	.001
Financial Judgments	1.81 (0.45)	1.61 (0.61)	1.94 (0.25)	1.71 (0.58)	1.68 (0.48)	.001

Table S2. Tukey's HDS *post-hoc* correction for multiple comparison. Significant *p-values* are reported in bold.

		Mean difference	Standard error	p-value	95% Confidence Interval	
					Lower bound	Upper bound
Counting currencies						
HC	MCI	.463	.106	**<.001***	.12	.81
	PD	.197	.121	.482	-.20	.59
	RS	.427	.147	**.031**	-.05	.91
	LS	.494	.183	.058	-.11	1.10
MCI	PD	-.267	.116	.147	-.65	.11
	RS	-.036	.143	.999	-.50	.43
	LS	.030	.180	1.000	-.56	.62
PD	RS	.231	.154	.566	-.28	.74

	LS	.297	.189	.518	-.32	.92
RS	LS	.067	.207	.998	-.61	.75
Reading abilities						
HC	MCI	.412	.099	**<.001***	.09	.74
	PD	.078	.113	.960	-.29	.45
	RS	.926	.137	**<.001***	.47	1.38
	LS	.533	.172	**.018**	-.03	1.10
MCI	PD	-.334	.108	**.019**	-.69	.02
	RS	.514	.134	**.001***	.08	.95
	LS	.121	.169	.952	-.43	.67
PD	RS	.848	.144	**<.001***	.37	1.32
	LS	.455	.177	.079	-.13	1.04
RS	LS	-.393	.194	.254	-1.03	.24
Item purchase						
HC	MCI	.369	.125	**.027**	-.04	.78
	PD	-.155	.142	.811	-.62	.31
	RS	.325	.173	.329	-.24	.89
	LS	.356	.216	.467	-.35	1.06
MCI	PD	-.524	.136	**.001***	-.97	-.08
	RS	-.044	.168	.999	-.60	.51
	LS	-.013	.212	1.000	-.71	.68
PD	RS	.480	.181	.064	-.12	1.08

	LS	.511	.223	.149	-.22	1.24
RS	LS	.031	.243	1.000	-.77	.83
Percentages						
HC	MCI	.726	.156	**<.001***	.21	1.24
	PD	.032	.178	1.000	-.55	.61
	RS	.539	.216	.094	-.17	1.25
	LS	1.116	.269	**<.001***	.23	2.00
MCI	PD	-.694	.170	**.001***	-1.25	-.14
	RS	-.187	.209	.899	-.88	.50
	LS	.390	.265	.580	-.48	1.26
PD	RS	.507	.226	.168	-.24	1.25
	LS	1.084	.278	**.001***	.17	2.00
RS	LS	.577	.304	.319	-.42	1.57
Financial Concepts						
HC	MCI	1.429	.230	**<.001***	.67	2.18
	PD	-.482	.262	.354	-1.34	.38
	RS	-.722	.318	.158	-1.77	.32
	LS	.789	.398	.277	-.52	2.10
MCI	PD	-1.911	.251	**<.001***	-2.74	-1.09
	RS	-2.151	.309	**<.001***	-3.17	-1.13
	LS	-.640	.391	.474	-1.92	.64
PD	RS	-.240	.334	.952	-1.34	.86

	LS	1.271	.411	**.018**	-.08	2.62
RS	LS	1.511	.449	**.008***	.04	2.98
Bill Payments						
HC	MCI	.332	.100	**.009***	.00	.66
	PD	-.056	.115	.988	-.43	.32
	RS	-.024	.139	1.000	-.48	.43
	LS	.160	.174	.888	-.41	.73
MCI	PD	-.388	.110	**.004***	-.75	-.03
	RS	-.356	.135	.068	-.80	.09
	LS	-.172	.171	.853	-.73	.39
PD	RS	.032	.146	.999	-.45	.51
	LS	.216	.180	.748	-.37	.81
RS	LS	.184	.196	.882	-.46	.83
Financial Judgments						
HC	MCI	.204	.073	**.046**	-.04	.44
	PD	-.126	.084	.562	-.40	.15
	RS	.104	.102	.847	-.23	.44
	LS	.125	.127	.862	-.29	.54
MCI	PD	-.330	.080	**<.001***	-.59	-.07
	RS	-.100	.099	.850	-.42	.22
	LS	-.078	.125	.970	-.49	.33
PD	RS	.230	.107	.202	-.12	.58

	LS	.251	.131	.312	-.18	.68
RS	LS	.022	.143	1.000	-.45	.49

**p-values* remained significant after Bonferroni correction; HC: Healthy controls; MCI: Mild Cognitive Impairment; PD: Parkinson's disease; RS: Right stroke; LS: left stroke
